# Laser-Induced Rehydration of Cryo-Landed Proteins Restores Native Structure

**DOI:** 10.1016/j.mcpro.2025.100987

**Published:** 2025-05-09

**Authors:** Keaton L. Mertz, Drew Jordahl, Colin A. Hemme, Mitchell D. Probasco, Dylan S. Forbes, Peter L. Ducos, Austin Z. Salome, Michael S. Westphall, Scott T. Quarmby, Timothy Grant, Joshua J. Coon

**Affiliations:** 1 Department of Chemistry, University of Wisconsin-Madison, Madison, Wisconsin, United States; 2 Department of Biomolecular Chemistry, University of Wisconsin-Madison, Madison, Wisconsin, United States; 3 Cellular and Molecular Biology Graduate Program, University of Wisconsin-Madison, Madison, Wisconsin, United States; 4 Department of Biochemistry, University of Wisconsin-Madison, Madison, Wisconsin, United States; 5 Morgridge Institute for Research, Madison, Wisconsin, United States

**Keywords:** cryo-electron microscopy, cryo-landing, soft landing, ion beam deposition, native MS

## Abstract

The use of native mass spectrometry (MS) to land biological molecules for subsequent cryogenic electron microscopy (cryoEM) imaging and three-dimensional reconstruction has gained momentum in recent years as a means to overcome long-standing challenges posed by traditional cryoEM sample preparation. However, recent results obtained with this approach have been constrained by low resolution and the compaction of cryo-landed particles, likely due to dehydration during exposure to vacuum. Here, we describe a new sample preparation method that uses a laser integrated into a cryogenic soft-landing apparatus to liquefy precisely deposited amorphous ice, rehydrating particles, and restoring their solution structure prior to rapid revitrification via the thermal mass of the grid. With this technique, we demonstrate the reconstruction of cryo-landed, rehydrated, and revitrified β-galactosidase that is comparable in resolution to that achieved with plunge freezing. Furthermore, these particles are not compacted, matching the known structure and conformation obtained with traditionally plunge-frozen particles. These results establish the viability of coupling native MS with cryoEM for high-resolution structural determination without the limitations imposed by conventional sample preparation, and they open a path to solving previously inaccessible molecules and to integrating MS capabilities such as gas-phase purification to complex samples such as cell lysates.

## Highlights

• Laser-induced rehydration restores native structure to cryo-landed proteins.
• This new method directly couples native MS with cryoEM for structural analysis.
• This novel technique resolves protein compaction from vacuum-induced dehydration.
• Rehydrated β-galactosidase particles show restored structure and no compaction.
• Resulting structures are comparable to conventional plunge freezing results.

## Graphical Abstract

**Figure:**
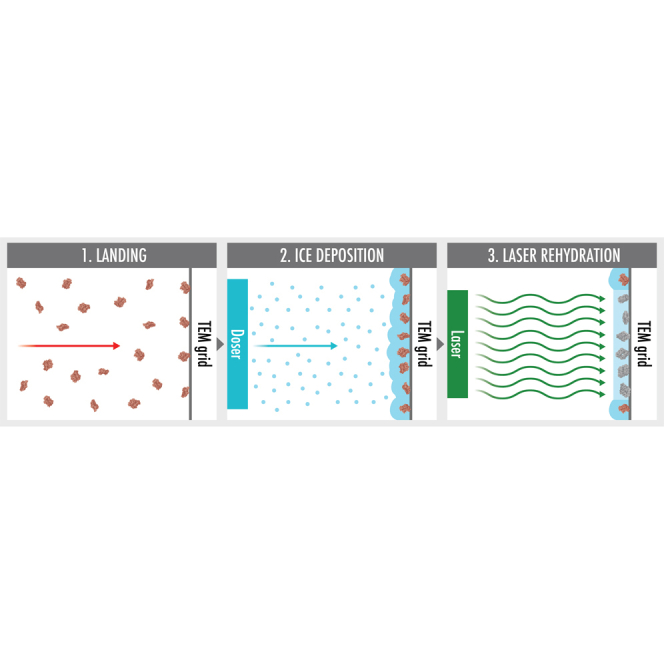

## In Brief

Landing biological molecules using native mass spectrometry (MS) has promise to overcome challenges with traditional cryogenic electron microscopy (cryoEM) sample preparation. However, particles are compacted likely due to dehydration in vacuum. Here, we use a laser to briefly liquefy the ice, rehydrating β-galactosidase particles and restoring their solution structure. Particles show comparable resolution to plunge freezing and show no evidence of compaction. These results establish the viability of native MS coupled to cryoEM for structural determination of previously inaccessible molecules.

Several laboratories have recently pursued new technologies that seek to utilize mass spectrometry (MS) to isolate and deposit biological macromolecules onto transmission electron microscopy (TEM) grids (1, 2, 3, 4, 5, 6, 7, 8, 9, 10, 11, 12). While early protein soft landing experiments (13) date back several decades to shortly after the introduction of electrospray ionization (14), the last several years have witnessed a renaissance of interest in this technique because it provides a path to eliminate the significant remaining challenges of conventional cryo-electron microscopy (cryoEM) (15) sample preparation (16). This approach, largely unchanged since its introduction nearly 50 years ago, relies on blotting solution samples into a thin layer with filter paper and then plunge freezing the cryoEM grid into liquid ethane to rapidly form vitreous ice (17, 18). Sitting in a thin film of aqueous solution for a relatively long time (seconds) causes particles to interact with the air–water interface thousands of times prior to vitrification (19). As a result, a significant number of samples are damaged or denatured. For those that survive, it is estimated that 90% of proteins are adsorbed to the air–water interface (20), where they often preferentially orient, limiting the quality of three-dimensional reconstruction.

The use of MS as a cryoEM landing technology has the potential to remove the air–water interface bottleneck (whilst offering MS sample isolation capabilities) and is in an intensive period of method development. For example, we have previously reported the use of chemical matrices and negative stain TEM to obtain three-dimensional structures of landed protein–protein complexes (7). More recently, we described an instrument that could land these complexes on cryogenically cooled TEM grids with subsequent deposition of amorphous ice *in vacuo* (10). These samples were directly imaged using cryoEM; however, despite having improved quality over room temperature-landed particles, they suffered from limited resolution, which we attributed to damage they had incurred prior to imaging. Subsequently, Rauschenbach *et al*. described a similar cryo-landing system, which they used to solve a high-resolution structure of β-galactosidase (11). This structure and molecular dynamic simulations enabled them to determine that the complex was significantly compacted due to dehydration caused by vacuum exposure.

Additional recent work has shown that this dehydration-driven compaction is sample dependent (21). That said, it is unclear exactly when in the cryo-landing process particles become compacted or if this compaction can be avoided. Given this, we hypothesized that a more practical solution would be to repair dehydrated, compacted particles post cryo-landing via rapid melting and revitrification (22). Working with traditionally plunge frozen grids, Lorenz *et al*. have demonstrated that irradiation with short laser pulses of visible light (532 nm) can briefly liquefy vitreous ice in vacuum (23, 24). If the laser spot size is small enough, the liquefied region is quickly revitrified owing to the thermal mass of the grid itself. We supposed that we could leverage this approach to rehydrate cryo-landed particles as a means for restoration of native structure (22).

Here we describe the incorporation of a molecular water doser for the precise deposition of amorphous ice and a 532 nm laser system for the subsequent liquefication of the ice to rehydrate cryo-landed particles into our previously described instrument (10). Using this system, we rehydrated cryo-landed β-galactosidase particles directly inside the landing chamber of the modified mass spectrometer. Unlike cryo-landed but not lasered particles imaged from the same grid, these laser-rehydrated particles demonstrate no appreciable compaction and are comparable to those prepared by conventional plunge freezing. We conclude that our laser rehydration strategy resolves the previously confounding issues of vacuum-induced dehydration and compaction and provides a direct route to coupling native MS with cryoEM for high-resolution reconstruction of native molecular structure.

## Experimental Procedures

### β-Galactosidase Preparation

β-Galactosidase was purchased from MilliporeSigma (*Escherichia coli*, G3153-5MG) and prepared at 1 mg/ml in 100 mM ammonium acetate. 50 μl of the protein solution was added to 450 μl of ammonium acetate and placed in a 100 kDa Amicon Ultra-0.5 centrifugal filter (MilliporeSigma). The sample was then centrifuged (Sorvall Legend Micro 21R, Thermo Fisher Scientific, Waltham, MA) at 12,500*g* for 5 min at 4 °C. The filtrate was discarded and the process repeated nine more times. To obtain the final sample, the filter was inverted and centrifuged at 2000*g* for 1 min and diluted with 100 mM ammonium acetate to a final concentration of 1 μM as quantified by UV/Vis using a Nanodrop One (1 Abs = 1 mg/ml, Thermo Fisher Scientific).

### Mass Spectrometry

Native static spray mass spectrometry experiments were performed on a modified Thermo Scientific Q-Exactive UHMR Hybrid Quadrupole-Orbitrap mass spectrometer (Thermo Fisher Scientific). For nano electrospray ionization (nanoESI), borosilicate glass capillary emitters were fabricated in-house using glass capillaries (B120–69–15, Sutter Instrument Company) and a micropipette puller (P-1000, Sutter Instrument Company) to achieve final orifice diameters of ∼2 to 10 μm. A platinum wire was inserted into the capillary emitter and placed in contact with the protein solution to apply a nanoESI voltage in the range of 1.1 to 1.5 kV. The emitter was situated 2 to 10 mm from the inlet of the mass spectrometer. Full-scan MS1 experiments were acquired by introducing nitrogen gas directly into the C-trap until the C-trap vacuum chamber pressure was 1 x 10^-4^ mbar. The identity of the β-galactosidase was confirmed using the Orbitrap mass analyzer at a resolving power of 6250 at 400 *m/z*, the inlet capillary temperature was set to 60 °C, and in-source trapping was set to 0 V (data not shown).

### Cryo-Landing Experiments

Proteins were cryo-landed onto R 2/2, 200 mesh UltrAuFoil grids (Quantifoil Micro Tools) coated with amorphous carbon. The carbon film was prepared by flash Joule heating carbon threads (ACE600, Leica Microsystems) and subsequently floated onto the UltrAuFoil grids which were then mounted into C-Clip Rings (1036173, Thermo Fisher Scientific). Prior to cryo-landing, up to four clipped grids were placed into grid holders and loaded into the cryo-landing apparatus vacuum chamber, which was heated overnight to 65 °C using three 10-W lamps. Before beginning experiments, the heating lamps were turned off and liquid nitrogen was used to cool the linear actuated cold probe (10). The probe temperature was recorded with a silicon diode temperature sensor embedded into the probe (DT-670, Lake Shore Cryotronics). The grid holder was then attached to the cold probe via a spring-loaded clamp and then cooled for 10 min at ∼1 mm distance from an aluminum surface previously desorbed of water by conducted heat from the nearby water doser which is heated to 120 °C. The chamber pressure was typically ∼5.3 × 10^−5^ mbar. Next, the gate valve connecting the landing apparatus to the vacuum chamber of the mass spectrometer was opened and the grid was then positioned 0.25 mm from the HCD exit lens. β-galactosidase ions were cryo-landed onto the grid for 25 min with an ion current of ∼12 pA as measured by a picoammeter (9103 USB Picoammeter, RBD Instruments).

During cryo-landing the flow of nitrogen to the C-trap was ceased. The C-trap chamber pressure thereby reduced to 8 × 10^−6^ mbar and the instrument was set to fragmentation mode with a collision energy of 1 V. This allowed ions to bypass both the Orbitrap analyzer and C-trap without stopping. A controlled DC potential gradient directed ions from the mass spectrometer inlet to the cooled grid with voltages decreasing from 70 V at the source DC offset down to −12 V at the grid. The specific settings were: source DC offset 70 V, injection flatapole 5 V, inter flatapole lens 5 V, bent flatapole 4 V, transfer multipole 3 V, C- trap entrance lens 2 V, HCD field gradient −5 V, HCD cell DC offset −8 V, HCD cell exit lens −11 V, and grid −12 V. Finally, a wide isolation window of 8000 to 12,000 *m/z* was utilized.

### Ice Deposition and Laser Rehydration

Following cryo-landing, the probe was retracted into the vacuum chamber of the landing apparatus and the gate valve was closed, isolating the landing apparatus from the mass spectrometer. Next, amorphous ice was deposited at a calibrated rate of 1.2 nm/s using a heated molecular water doser positioned 6 mm from the grid surface, ensuring uniform coverage. The ice deposition growth rate was initially measured by a quartz crystal microbalance (10). The molecular water doser was heated to 120 °C by a 15-W resistive heater with the temperature readback by a platinum resistance temperature sensor both press-fit into the brass housing of the dosing unit.

After ice deposition, a prism and lens assembly were used to deliver focused laser pulses to the grid surface. A continuous-wave 532 nm laser (Laser Quantum gem 532 FS CW laser head, Sterling, VA) was modulated into 15 μs pulses using an acousto-optic modulator (AOM, 4215–6, Gooch & Housego) driven by a voltage-controlled oscillator (VCO, MAX2870 module, Analog Devices). The VCO is chopped by an RF switch (ZFSWA2-63DR+, Mini-Circuits) and amplified (ZJL-6G+ and ZHL-2010+, Mini-Circuits) before reaching the AOM. The modulator’s frequency was tuned from 118 to 136 MHz, translating to laser beam positioning across the surface of the grid of 38 μm per MHz frequency change. Laser power was maintained and operated at 100 to 175 mW as reported by the internal photodiode of the laser head. This corresponds to 15 to 26 mW at the grid as measured by a silicon diode power meter (DET100A2, Thorlabs). The focused laser beam (∼20 μm FWHM) was systematically, rectangularly rastered across the grid using a stepper motor-driven linear stage with 0.10-mm increments (x-axis) and 0.057-mm AOM increments (y-axis).

Upon completion of laser exposure, the grid holder was released from the cryogenically cooled probe and dropped onto the lower valve of a vacuum interlock comprising two stacked pneumatically driven gate valves. The top gate valve was then closed, thereby isolating the grid from the cryo-landing apparatus vacuum chamber. The lower gate valve was then immediately opened, which rapidly vents the interlock region to a pressure of ∼1 atm of nitrogen drawn from the headspace of a liquid nitrogen Dewar. The grid drops out of the lower gate valve into an insulated cup of liquid nitrogen. The total transfer is computer-controlled and completed in under a second.

### CryoEM Imaging and Data Processing

Cryo-landed grids were inserted into a Thermo Scientific 200 kV Glacios microscope (Thermo Fisher Scientific) for imaging. Movies of the cryo-landed grids were collected at 73,000×, with a nominal pixel size of 1.945 Å using EPU. 476 movies were collected for cryo-landed and laser-rehydrated β-galactosidase in ice (∼20 nm), followed by 2454 movies for cryo-landed and non-lasered β-galactosidase on the same grid. 2474 standard plunge-frozen β-galactosidase movies were collected at 120,000× with a nominal pixel size of 1.200 Å. All data were collected using a Falcon 3EC direct electron detector operated in integrating mode and a defocus range of 0.5 to 2.0 μm. The cryo-landed data were collected with a total dose of 60 e/Å^2^ over 84 frames. The plunge-frozen data were collected with a total dose of 36 e/Å^2^ over 33 frames.

Collected movies were imported into *cis*TEM 2.0 for processing (25). Movies were processed following the standard workflow of motion correction, exposure filtering (26), and contrast transfer function (CTF) estimation (27).

Micrographs with poor CTF fit resolution and micrographs where the ice was crystalline were removed. Automated particle picking and then 2D classification within *cis*TEM were utilized to select the best particles in all datasets. An ab-initio starting reference was then created from the best class averages, after which those same particles were used in 3D auto-refinement. The final particle count was 5684 for the cryo-landed and laser-rehydrated β-galactosidase, 138,063 particles for the cryo-landed and non-lasered β-galactosidase, and 54,258 particles for the standard plunge-frozen data. However, to make volumes comparable for the comparisons shown in this study, in all cases only 5684 particles were used. D2 symmetry was imposed during all refinement steps. To make the angular distribution maps comparable, the final particles were all aligned with the same reference. The highest resolution information included in the 3D refinement steps was 20 Å for all reconstructions.

As we were primarily interested in each reconstruction’s resolution compared to the known structure, we used map-to-model Fourier shell correlations (FSCs) calculated against PDB structure 6CVM (28, 29) to measure the resolution of the reconstructions. The reconstructions were pre-masked with a shaped 3D mask based upon the PDB structure and the FSC was calculated against a map of 6CVM simulated in ChimeraX (30) at 2 Å resolution. Reported resolutions were obtained from the resulting curves using an FSC cut-off threshold of 0.5 (supplemental Fig. S1). The measured resolutions were as follows: 8.2 Å for the cryo-landed and laser-rehydrated β-galactosidase, 20 Å for the cryo-landed and non-lasered β-galactosidase, and 5.9 Å for standard plunge-frozen β-galactosidase.

## Results

### Construction and Use of Integrated Laser Melting Apparatus

**Fig. 1:**
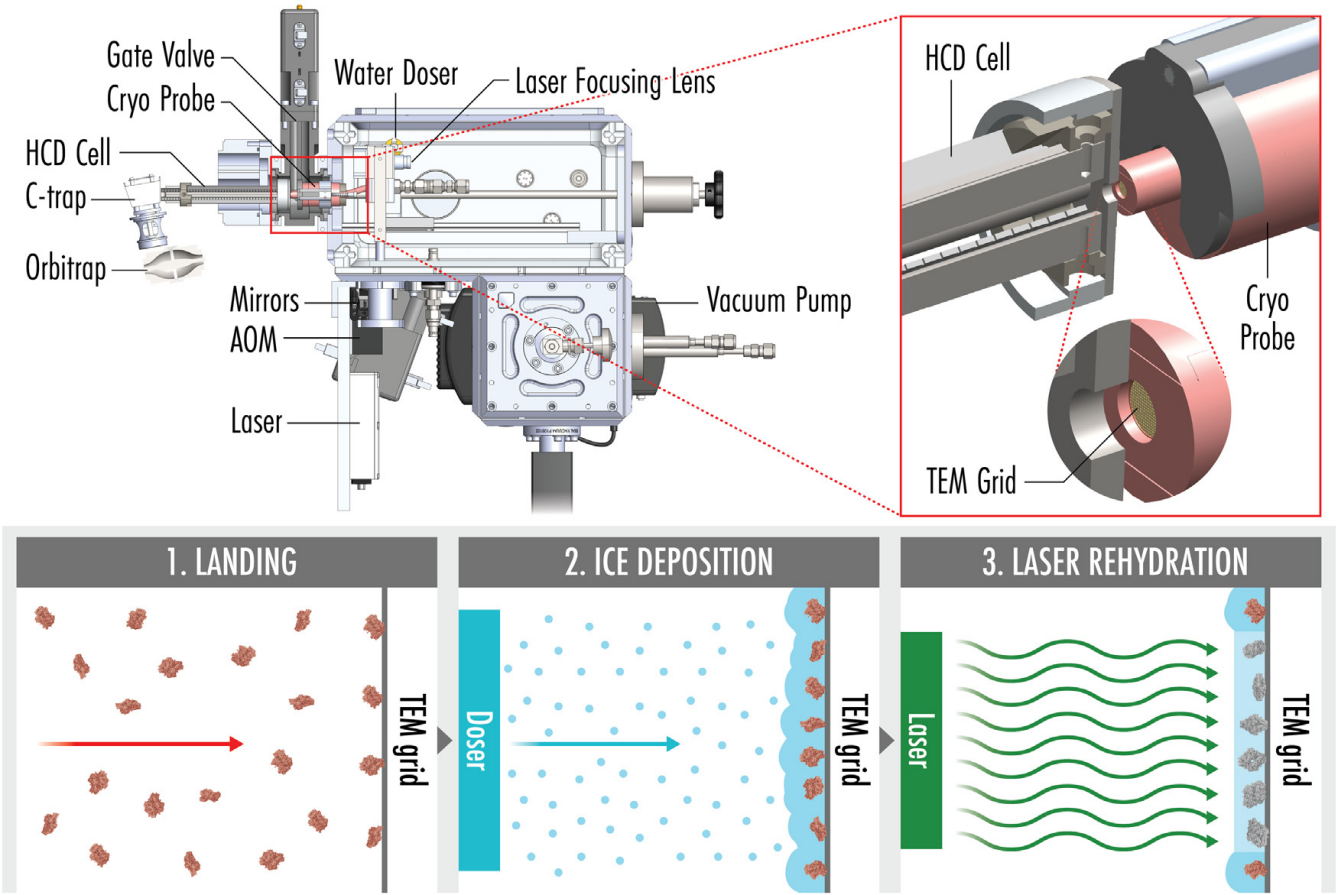
**Overview of cryo-landing, ice deposition, and laser rehydration technique.** The schematic in the upper *left* portion shows a mechanical drawing of the cryo-landing instrument described herein. Shown in the inset is the cryo probe holding a TEM grid in the landing position. The *bottom* panels depict the (1) deposition of particles onto the cryogenically cooled grid, (2) the formation of amorphous ice using the molecular water doser, and (3) laser rehydration using a pulse of 532 nm light.

To rehydrate cryo-landed particles, we built upon our previously reported cryo-landing device (10) and integrated a 532 nm laser irradiation system (22). This wavelength has previously been demonstrated by Lorenz *et al*. to liquefy ice without damaging the particles and allowing revitrification via the thermal mass of the grid (23). Figure 1 depicts a schematic of our new device wherein we (1) deposit biomolecular cations onto the cryogenically cooled TEM grid, (2) precisely deposit the desired thickness of amorphous ice using a molecular water doser, and (3) irradiate the grid with the integrated laser. This setup can deliver 1.5 to 100 mW to the grid surface with a focused spot size of ∼20 μm FWHM. Furthermore, an acousto-optic modulator (AOM) allows precise timing of beam pulse width and beam rastering (in one dimension). By mounting the integrated water beam doser, laser mirror, and focusing lens on a stepper motor-controlled translation stage, we achieve precise computer control of amorphous ice growth and laser beam rastering in the dimension orthogonal to the AOM rastering.

**Fig. 2:**
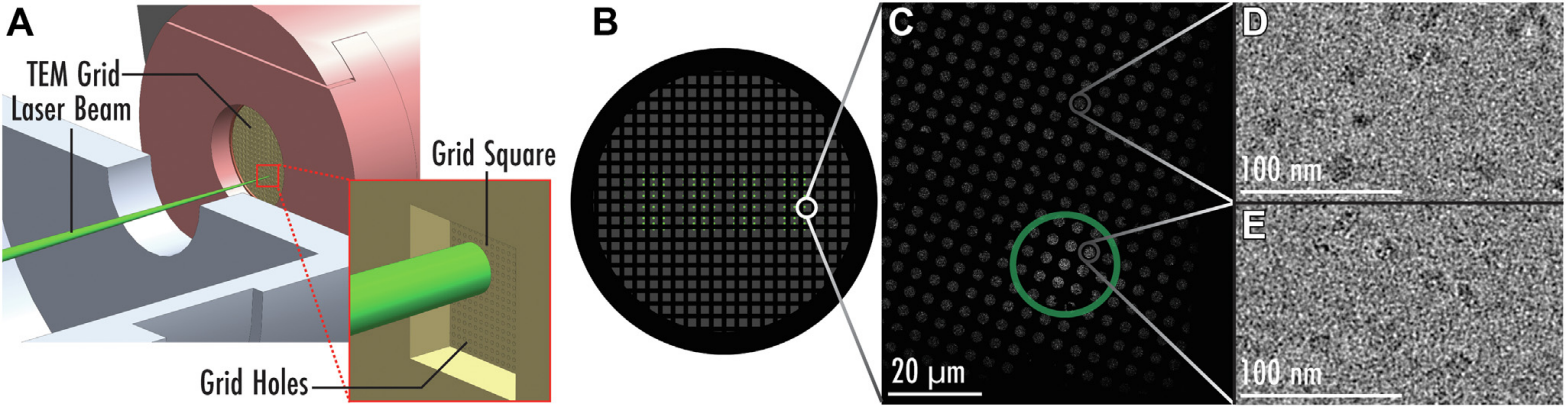
**Laser rehydration overview and application on cryo-landed β-galactosidase**. *A*, a close-up of the laser rehydration process showing the proportion of the laser spot to an individual *grid square*. *B*, the laser rastering pattern produced using the stepper motor and AOM for x and y translation, respectively. *C*, a zoom-in view of one grid square demonstrating an actual laser spot (*green circle*) covering ∼15 grid foil holes. Representative images of β-galactosidase taken from outside (*D*) and inside (*E*) the laser spot.

Figure 2*A* presents a cross-sectional view of the focused laser striking a TEM grid held in the cryo-landing probe and conveys the scale of the 20 μm irradiation zone in the context of the grid square (200 mesh, 80 μm square, 2 μm holes). A schematic of the laser spot positions used in this study are shown in Figure 2*B*. Note at present there is no alignment of the laser raster pattern with the grid mesh so that some laser spots are not usable as they strike grid bars. A zoom-in view of an actual laser spot as imaged on a grid is shown in Figure 2*C*. The spot is visible as a brighter region demonstrating some evaporation of water has taken place, presumably during liquefication. Representative images of cryo-landed β-galactosidase particles taken outside and inside of the area melted by the laser spot are shown in Figure 2, *D* and *E*, respectively.

### Laser-Induced Rehydration of β-Galactosidase

**Fig. 3:**
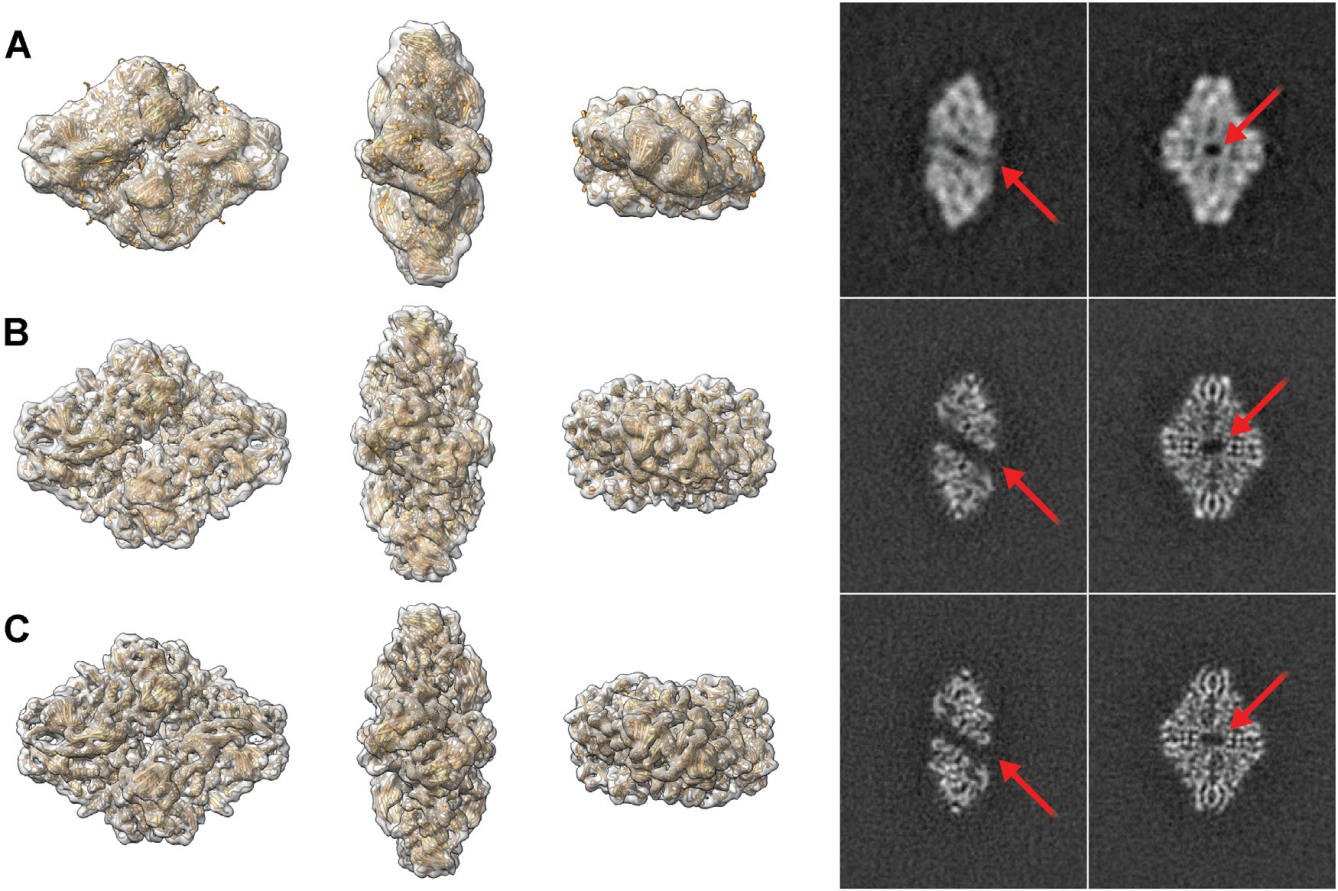
**Comparison of β-galactosidase reconstructions.***Left*: PDB:6CVM fit into density maps calculated from ∼5000 particles from (*A*) cryo-landed particles; (*B*) cryo-landed and laser rehydrated particles (5,000); and (*C*) conventionally plunge-frozen particles. The map-to-model resolutions of these reconstructions are 20.0, 8.2, and 5.9 Å, respectively. *Right*: selected two-dimensional slices from the corresponding density maps. The cryo-landed maps demonstrate previously observed compaction as compared to the cryo-landed and laser-rehydrated and plunge-frozen density maps which match known structure (see red arrows in panel A versus panels B and C).

To assess the effect of laser rehydration on particle compaction, we collected cryoEM datasets of β-galactosidase particles from both inside and outside laser spots from the grid shown above. Subsequent processing of these datasets resulted in three-dimensional reconstructions, which are shown in Figure 3. The reconstruction from particles not subjected to laser rehydration (*i.e.*, outside) appears very similar to our previously published structures (10), demonstrating a low map-to-model resolution of 20.0 Å (Fig. 3*A*). By contrast, the reconstruction obtained from particles exposed to the laser spot (*i.e.*, inside) has a map-to-model resolution of 8.2 Å (Fig. 3*B*). For comparison, we show a reconstruction resulting from a traditionally plunge-frozen grid (Fig. 3*C*) collected with the same number of particles on the same microscope. The lacey carbon grid used in this case was covered in a significantly thinner layer of amorphous carbon than the cryo-landed grid, which would be expected to lead to better quality data. This reconstruction has a calculated map-to-model resolution of 5.9 Å. To illustrate the difference in compaction, we extracted slices from each of these reconstructions (Fig. 3, right column). The slices extracted from the cryo-landed but not lasered reconstruction demonstrate clear compaction (note red arrows in Fig. 3*A*), whereas the slices extracted from the laser rehydrated and plunge-frozen reconstructions are similar and uncompacted, matching the true, known structure.

Considering both the low number of particles (∼5000) and the older generation cryoEM direct electron detector (Falcon 3EC), these results are consistent with our expectations. Additionally, the plunge-frozen data was taken on a grid with a significantly lower noise background due to the use of thinner amorphous carbon. Also, the plunge frozen particles were a random selection of the particles available after fully processing the ∼54,000 available, whereas for the cryo-landed and laser rehydrated particles, all particles that were available were used. We therefore believe the results to be comparable.

We next compared the angular distributions obtained from the different datasets (supplemental Fig. S2) to assess preferential orientation. Here we found that the cryo-landed particles exhibit a wider range of views compared to the plunge frozen sample. Interestingly, the particles from within the laser-induced rehydration spots exhibit more preferential orientation than those not laser rehydrated. We attribute this to the fact that we deposited an exceptionally thin ice layer on the grid (∼20 nm outside the laser spots and ∼17 nm inside after evaporation), a thickness which would be almost impossible to achieve routinely during conventional plunge freezing (31). However, the longest dimension of β-galactosidase is ∼18 nm (32) and therefore the particles were likely more constrained within this thin ice layer. These data suggest the need to calibrate ice thickness to particle size while accounting for evaporative loss during liquefication.

## Discussion

Multiple publications have established that dehydration of biomolecular cations during cryo-landing leads to structural compaction (10, 11, 21). CryoEM reconstructions solved from such particles, therefore, do not describe biologically relevant structures, reducing the potential utility of the technology. We demonstrate here that such damage can be overcome by rehydrating the cryo-landed particles. We achieve this by depositing amorphous ice onto cryo-landed particles and subsequently using a laser pulse to rapidly liquefy regions of ice, thereby rehydrating the particles contained within them before the thermal mass of the grid quickly revitrifies the area. In the current implementation, the water doser and laser are installed directly into the cryo-landing apparatus such that all steps are performed in place. The pulse power, duration, and location of laser irradiation can be precisely controlled, supporting different grid configurations. Using this technology, we have demonstrated β-galactosidase reconstructions that are comparable to those prepared by conventional plunge freezing. This essential initial benchmark opens a direct path to combine MS cryo-landing and cryoEM analysis to propel structural biology.

CryoEM samples prepared using this technology have many potential benefits. Precise control of the thickness of the deposited ice layer allows for particles to be embedded in layers thinner than can be achieved via conventional plunge freezing. This will lead to significant improvements in contrast, particularly in the case of small particles (31). Above all, plunge freezing leads to damage and/or orientation bias caused by interactions at the air–water interface (20, 33, 34, 35). Previous studies have demonstrated that shorter exposure (50–100 ms) to this interface can reduce these effects (36, 37, 38, 39). The liquefication induced by the laser pulse is only ∼10 μs (40), or 3 orders of magnitude shorter, potentially drastically reducing or even eliminating this problem.

A current limitation of our method is the low density of laser spots (15 laser spots covering 476 grid foil holes) that limit the reconstructions we present here to only a few thousand particles. To routinely solve high-resolution structures, we will need to improve the number of laser rehydrated particles present on each grid from the current ∼5000. The primary obstacle is that in our current laser rehydration setup, our laser spots hit imageable areas (*i.e.* open grid squares) only ∼20% of the time and our rastering coverage is limited to only one third of the grid. We estimate that our planned next generation system, which has precise guiding of laser spots to grid squares and can automatically raster the entirety of the grid, will substantially boost the rehydrated particles on a single grid by ∼40 fold. Further, as mentioned above, despite having a very thin ice layer, the grids used here contained a thick layer of amorphous carbon, which generated significant background noise. Future experiments could be improved by using a thinner support layer, *e.g.* ultraclean monolayer graphene, which should provide essentially no background.

If these challenges can be overcome, we anticipate this approach will enable the creation of ideal cryoEM samples. For example, by consistently matching the thickness of the deposited ice layer to the specific protein size, all grids will have optimal image contrast. This cannot be achieved for small proteins (<20 nm) with conventional plunge freezing (31). Additionally, many samples are drastically affected by the air–water interface, with many degraded to the point where a structure cannot be obtained. The likely decrease in air–water interface effects with our method may overcome these barriers. These benefits can be further amplified by the ability of the mass spectrometer to purify and characterize the particles of choice from much more complicated mixtures than is currently possible.

We conclude such capabilities will open new frontiers and experiments that were previously beyond reach. For example, one could imagine using varying laser pulse durations to study dynamic protein–protein interactions by sequentially landing interaction partners and imaging them in a precise time-lapsed sequence. Finally, these results open a path to solving previously inaccessible molecules and to integrating MS capabilities such as gas-phase purification to complex samples such as cell lysates. Specifically, chromatographic methods (*e.g.*, size-exclusion chromatography) already exist for separating intact protein complexes from cell lysate (41), and with the purification capabilities of mass spectrometry, the effluent of these separations could be directed into the cryo-landing apparatus whereby they are further purified, characterized, and landed for high-throughput cryo-EM imaging.

## Data Availability

β-galactosidase density maps, structures, and FSC curves for cryo-landed, cryo-landed then laser rehydrated, and pipetted then plunge frozen samples have been deposited in the Electron Microscopy Data Bank under accession codes EMD-70551, EMD-70549, and EMD-70552, respectively.

## Supplemental data

This article contains supplemental data.

## Conflict of interest

The authors declare the following financial interests/personal relationships which may be considered as potential competing interests: J. J. C. is a consultant for Thermo Fisher Scientific and Seer. J. J. C. is a co-founder of CeleramAb Inc.
