# Supplementary material for: Laser-Induced Rehydration of Cryo-Landed Proteins Restores Native Structure
Source: Mol Cell Proteomics. 2025 May 9;24(6):100987. doi: 10.1016/j.mcpro.2025.100987 (PMC12171540; doi:10.1016/j.mcpro.2025.100987)
Supplement: Supplemental data [file mmc1.docx]

**SUPPORTING INFORMATION**

**Laser-Induced Rehydration of Cryo-Landed Proteins Restores Native Structure**

Keaton L. Mertz^1,2†^, Drew Jordahl^2,3†^, Colin A. Hemme^4,5^, Mitchell D. Probasco^5^, Dylan S. Forbes^1,2^, Peter L. Ducos^4,5^, Austin Z. Salome^1,2^, Michael S. Westphall^2^, Scott T. Quarmby^2^, Timothy Grant^4,5^*, and Joshua J. Coon^1,2,5^*

^1^Department of Chemistry, University of Wisconsin-Madison, Madison, Wisconsin 53706, United States

^2^Department of Biomolecular Chemistry, University of Wisconsin-Madison, Madison, Wisconsin 53706, United States

^3^Cellular and Molecular Biology Graduate Program, University of Wisconsin-Madison, Madison, Wisconsin 53706, United States

^4^Department of Biochemistry, University of Wisconsin-Madison, Madison, Wisconsin 53706, United States

^5^Morgridge Institute for Research, Madison, Wisconsin 53515, United States

*^†^These authors contributed equally*

**Correspondence to* [*tgrant@morgridge.org*](mailto:tgrant@morgridge.org) *or* [*coon@wisc.edu*](mailto:coon@wisc.edu)

**SUPPLEMENTARY FIGURES**

**
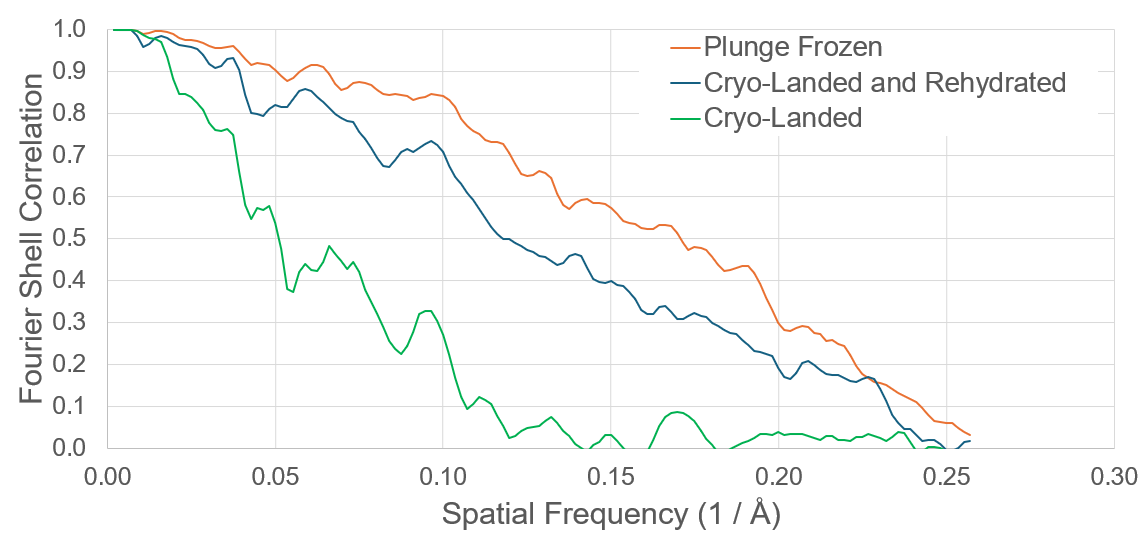
**

**Supplementary Figure 1. Map-to-model Fourier shell correlations** for conventionally plunge-frozen particles, cryo-landed and laser rehydrated particles with thin ice, and cryo-landed particles.

**
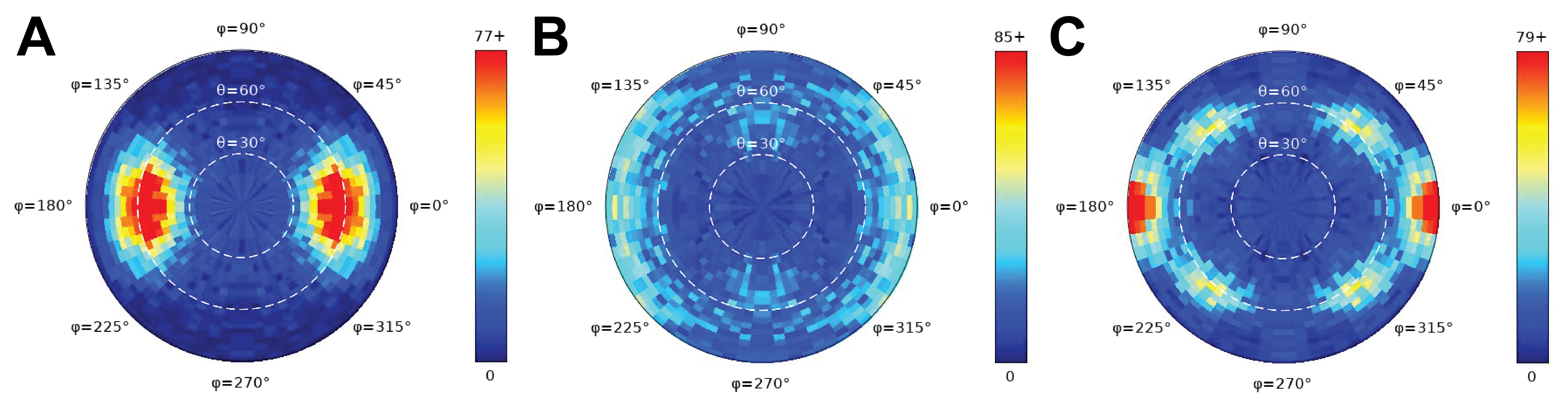
**

Supplementary Figure 2. Angular distribution maps illustrating populated views for (A) conventionally plunge-frozen particles; (B) cryo-landed particles; (C) cryo-landed and laser rehydrated particles with thin ice. Cryo-landed particles exhibit much less preferential orientation than conventionally plunge-frozen samples. Laser-rehydrated particles in thin ice show preferential orientation, an observation that we attribute to the thin ice being slightly thinner (17 nm) than the β-galactosidase particles are tall (18 nm).
